# Identification of human pregnane X receptor antagonists utilizing a high-throughput screening platform

**DOI:** 10.3389/fphar.2024.1448744

**Published:** 2024-10-23

**Authors:** Caitlin Lynch, Ryan Margolis, Jacob Niebler, Jameson Travers, Srilatha Sakamuru, Tongan Zhao, Carleen Klumpp-Thomas, Ruili Huang, Menghang Xia

**Affiliations:** National Center for Advancing Translational Sciences, National Institutes of Health, Bethesda, MD, United States

**Keywords:** antagonist, drug metabolizing enzymes, fusidic acid, gamma-secretase modulator 2, high-throughput screening, pregnane X receptor

## Abstract

Pregnane X receptor (PXR) is a xenobiotic-sensing nuclear receptor with a well-established role in regulating drug metabolism and clearance. Recent studies have shown that PXR is involved in cell proliferation, apoptosis, immune response, and energy homeostasis. It is important to identify compounds that may modulate PXR activity to prevent drug-drug interactions, distinguish chemicals which could potentially generate toxicity, and identify compounds for further development towards therapeutic usage. In this study, we have screened the National Center for Advancing Translational Sciences (NCATS) Pharmacologically Active Chemical Toolbox (NPACT) library, which consists of 5,099 unique pharmacologically active synthetic and naturally derived small molecules to identify PXR antagonists. Ninety-four compounds were identified as potential PXR antagonists through a primary screen and 66 were confirmed in a confirmation study. Of these compounds, twenty potential PXR antagonists, including gamma-secretase modulator 2 (GSM2) and fusidic acid, were selected for further study based on their efficacy, potency, and novelty. Their PXR inhibition abilities were assessed by examining their effects on cytrochrome P450 (CYP) 3A4 mRNA expression using metabolically competent HepaRG cells. Additionally, a pharmacological inhibition assay using various concentrations of rifampicin as a stimulator was performed in HepG2-*CYP3A4*-hPXR cells to confirm the activity of the 20 selected compounds against PXR. Finally, HepaRG cells were used to confirm PXR antagonism by verification of a concentration-dependent decrease of CYP3A4 when co-treated with the known PXR agonist, rifampicin. Additionally, the potent actives were further investigated using molecular docking to find the potential interactions of the novel ligands with the active sites of hPXR. To our knowledge from the current study, GSM2 and fusidic acid have been identified as novel PXR antagonists, which provides useful information for further investigation regarding possible drug-drug interactions, as well as the detection of potential therapeutic effects or other toxic consequences.

## 1 Introduction

The pregnane X receptor (PXR) is a nuclear receptor that is known to play a significant role in the metabolism of many drugs and other xenobiotic compounds, as well as in cell proliferation, apoptosis, immune response, and energy homeostasis (Pondugula et al., 2016; Mackowiak et al., 2018). PXR, found predominantly in the liver and intestines (He et al., 2013), regulates drug metabolism primarily through its role as a transcription factor for cytochrome P450 3A4 (CYP3A4), which is responsible for the metabolism of around 50% of all clinical drugs (Lynch, et al., 2019). PXR in its inactive state is localized to the cytosol. However, activation of PXR, by ligand-binding, results in translocation to the nucleus, where binding to response elements within a *CYP3A4* promoter region facilitates transcription (Mackowiak et al., 2018, Dutta, Lim et al., 2022). While this activity is augmented through the inclusion of co-activators, such as transcriptional mediator/intermediary factor 2 (TIF2) and steroid receptor coactivator 1 (SRC-1), it is repressed through co-repressors, such as silencing mediator of retinoic acid and thyroid hormone receptor (SMRT) and nuclear receptor corepressor (NCoR) (Oladimeji, Cui et al., 2016).

Analysis of a wide range of potential modulators of PXR activity is very important due to the significance of its activation in altering expression of CYP3A4, as well as other drug metabolizing enzymes (DMEs). Altered expression of these DMEs can result in drug-drug, drug-herbal, or drug-environment interactions which may impact therapeutic efficacy or lead to toxicity of either entity (Mackowiak et al., 2018). Furthermore, PXR has a large ligand-binding domain which is capable of binding to various compounds with low specificity (Mackowiak et al., 2018, Dutta, Lim et al., 2022). It is therefore exceedingly important to identify the wide range of compounds that may bind to PXR and subsequently alter DME expression.

PXR, which is also known to be expressed differentially in multiple cancer types including liver, prostate, breast, and colon, regulates expression of many other genes involved in cell proliferation, metastasis, apoptosis, and energy homeostasis within cancer cells (Pondugula et al., 2016). Expression level of PXR can therefore impact cancer growth and progression, as well as chemotherapy effectiveness due to the role of CYP3A4 and other DMEs in metabolizing chemotherapeutic agents that may be used in treatment. Previous studies displayed PXR activation playing a key role in cancer drug resistance (Gupta et al., 2008; Mani et al., 2013) as well as conflicting evidence about PXR agonists causing tumor aggressiveness (Wang et al., 2011; Mani et al., 2013; Pondugula et al., 2016). Another study established that PXR has a complimentary role alongside aryl hydrocarbon receptor (AHR) inducing DNA damage after exposure of skin to the tumor initiator 7,12-dimethylbenz(a)anthracene (DMBA). However, Pxr^−/−^ mice protected epidermal cells from DNA damage (Elentner et al., 2015). This implies that a PXR antagonist could potentially be used therapeutically to protect the skin from DNA damage illustrating another cause for PXR modulator identification.

Immune response has also been demonstrated to be altered by modulation of PXR. Dubrac et al. discovered that pharmaceutical activation of PXR led to an inhibition of T lymphocyte function through inhibiting CD25, a T lymphocyte activation marker (Dubrac et al., 2010). These studies indicate the potential for PXR agonists to be used as immunosuppressants (Dubrac et al., 2010; Mackowiak et al., 2018). To determine if these PXR agonists can be used therapeutically, it would be revealing to have a PXR antagonist to use in contrast and determine true PXR involvement. Alongside the testing implications, beneficial PXR antagonists will need to go through further evaluation to elucidate their inhibition of the immune response. A more targeted or personalized method will likely be necessary if a PXR antagonist is to be used therapeutically.

As stated previously, PXR has recently been implicated as being a therapeutic target, meaning identifying a PXR antagonist is paramount for this research to continue. In 2001, ET-743 was the first PXR antagonist to be reported, followed by numerous others including metformin, ketoconazole, coumestrol, et cetera. However, many of these compounds are not therapeutically relevant as they are not potent enough to antagonize PXR without sufficient toxicities occurring *in vivo* (Mani et al., 2013). Through biochemical, cellular, and *in vivo* characterization, Lin et al. identified SPA70 as a highly selective and potent PXR antagonist with minimal toxicity (Lin et al., 2017). This important molecule recruits co-repressors to suppress hPXR activity, while also interacting with the AF-2 helix within the ligand binding domain. To our knowledge, this is the preeminent representative of a hPXR antagonist which can be used as an accurate pharmacological tool and potentially should be studied as a therapeutic treatment. However, it is novel and will, therefore, require years of study and research to be used therapeutically. Identifying another potent and selective hPXR antagonist, which is already a therapeutically used compound, would be intriguing and important research.

It is well established that PXR is one of the most important nuclear receptors when it comes to drug metabolism and drug-drug interactions; however, recently it has also been determined to have an impactful role in cancer, immune response, and energy homeostasis. As a result, these factors suggest it is necessary to identify compounds which can inhibit this diverse nuclear receptor. In this study, we screened the NCATS Pharmacologically Active Chemical Toolbox (NPACT) compound library, which consists of 5,099 unique compounds, at multiple concentrations, using HepG2 cells, an immortalized hepatocellular carcinoma cell line, stably transfected with hPXR, to discover PXR antagonists. To confirm these potential PXR antagonists, we have used a pharmacological IC_50_ shift study as well as HepaRG cells to examine CYP3A4 mRNA inhibition. Utilizing this method, we have identified gamma-secretase modulator 2 (GSM2) and fusidic acid as PXR antagonists that need further study to determine the extent of therapeutic usage or toxic side effects of these compounds in the human body. Fusidic acid, GSM2, and the other potential antagonists were further evaluated by docking them to the crystal structure of the hPXR to confirm their interactions with the active site.

## 2 Materials and methods

### 2.1 NPACT compound library

The NPACT compound library consists of more than 5,000 compounds, including naturally occurring and synthetically derived pharmacologically active small molecules. Among these compounds are all current quality-approved or investigational drugs, as well as tool compounds that may provide useful structural information in clinical drug development and valuable insight into different biological mechanisms or pathways.

### 2.2 Cell culture

HepG2 cells stably transfected with a *CYP3A4*-luc promoter construct and hPXR expression plasmid were provided by Dr. Taosheng Chen (Department of Chemical Biology and Therapeutics, St. Jude Children’s Research Hospital) (Lin et al., 2008). Cells were thawed in EMEM (ATCC, Rockville, Maryland), supplemented with 10% Hyclone FBS (GE Healthcare Life Sciences, Logan, Utah) and 100 U/mL of Penicillin and 100 μg/mL of Streptomycin (Invitrogen, Carlsbad, CA) and kept in an incubator at 37°C and 5% CO_2_. To culture these HepG2-*CYP3A4*-hPXR cells, 500 μg/mL of Geneticin (GIBCO, Gaithersburg, MD) was added to the thawing media.

### 2.3 PXR luciferase reporter gene assay

HepG2-*CYP3A4*-hPXR cells were dispensed into tissue culture treated 1536-well white assay plates (Greiner Bio-One North America, Monroe, NC) using a Multidrop Combi (Thermo Fisher Scientific Inc., Waltham, Massachusetts) at 2,500 cells/4 µL/well. The cell suspension was prepared using new assay media, which contained phenol red-free DMEM (GIBCO), 5% charcoal/dextran-treated FBS (Invitrogen), 1 mM sodium pyruvate (Invitrogen), 2 mM L-Glutamine (Invitrogen), and 100 U/mL of Penicillin and 100 μg/mL of Streptomycin (Invitrogen). All plates were then incubated for 4 h in a 37°C and 5% CO_2_ incubator. Following the incubation period, 23 nL of test compounds dissolved in dimethyl sulfoxide (DMSO), the positive control SPA70 (4-(4-tert-Butylphenylsulfonyl)-1-(2,5-dimethoxyphenyl)-5-methyl-1H-1,2,3-triazole), tetraoctylammonium bromide (the positive control for cytotoxicity), or vehicle control (DMSO) was added to each well using a Wako Pintool Station (Wako Automation, San Diego, California). In the primary screen, each compound was tested at 7 concentrations ranging from 46 μM to 1.5 nM. 1 μL of rifampicin, a known PXR agonist, was also co-treated in each well, generating a final concentration of 2 μM, using a Flying Reagent Dispenser (Aurora Discovery, Carlsbad, California). All assay plates were placed in an incubator at 37°C with 5% CO_2_ for 23 h. Following the incubation period, 1 µL of CellTiter-Fluor reagent (Promega, Madison, Wisconsin) was added to each well and all assay plates were placed back into the incubator set at 37°C and 5% CO_2_ for 1 h. Fluorescence intensity at 540 nm was measured following excitation at 405 nm using a ViewLux plate reader (Perkin Elmer, Shelton, Connecticut), in order to assess cell viability following treatment. Directly after the fluorescent read, 4 μL of ONE-Glo Luciferase reagent (Promega, Madison, Wisconsin) was added to each well using a Flying Reagent Dispenser, and plates were left to sit at room temperature for 30 min. The luminescence of each plate was then measured using the ViewLux plate reader and data were expressed as relative luminescence units. Gross assay performance was assessed by calculating quality metrics, such as coefficient of variance (CV), signal-to-background ratio (S/B), and Z′ factor, a measure of statistical effect size frequently used to judge whether an assay can be scaled up to a large high-throughput screen (Zhang et al., 1999).

### 2.4 Data analysis

Data analysis was completed using a previously described protocol (Huang et al., 2016). All plate reads were normalized to the positive control (SPA70) and vehicle control (DMSO) reads, where SPA70 (a PXR antagonist, 10 μM) was set as −100% activity, and DMSO was set as 0% activity. Normalized percent activity for all test compounds was determined using the following equation: % activity = [(*V*_compound_ - *V*_DMSO_)/(*V*_DMSO_–*V*_pos_)] × 100, where *V*_compound_ denotes the compound well values, *V*_pos_ denotes the median value of the positive control wells, and *V*_DMSO_ denotes the median value of the DMSO-only wells. Following normalization, data points were corrected using DMSO-only plates included at the beginning and end of the compound plate stack using an in-house pattern correction algorithm in order to remove background patterns, as well as other small abnormalities within the dataset (Southall et al., 2009). Efficacy (maximum response) and the half maximum inhibition (IC_50_) values for each compound were calculated by fitting concentration-response curves to a four-parameter Hill equation (below) (Wang et al., 2010), where *a* is % activity, *c* is compound concentration, *a*_*0*_ is activity at zero concentration, *a*_*inf*_ is activity at infinite concentration, *k* is IC_50_, and *n* is the Hill coefficient. Efficacy is then [*a*_*inf*_ - *a*_*0*_].

$$a=a_{0}+\left(a_{\inf}-a_{0}\right)\;\frac{c^{n}}{k^{n}+c^{n}}$$

Corresponding to the type of concentration-response curve detected, each compound was assigned as Class 1–4 based on efficacy, the number of data points observed above background activity, and the quality of fit (Wang et al., 2010; Huang et al., 2011). Curves with multiple points of activity are assigned Class 1 (complete curve with two asymptotes) or 2 (incomplete curve with one asymptote), which are further divided into subclasses based on efficacy and quality of fit; curves with a single point of activity are designated Class 3; and Class 4 curves are inactive. Curve class was further converted to curve rank by combining each class with efficacy, such that good quality, efficacious curves are assigned large absolute curve rank values. Curve rank is a numeric measure of compound activity ranging from −9 to 9, where a large negative value indicates a strong inhibitor, a large positive value denotes a strong activator, and 0 means inactive (Huang et al., 2016). The activity outcome was based on the PXR antagonist readout activity and a cell viability counter screen. Compounds with a curve rank < -1, efficacy < -50%, and inactive in the cell viability counter screen or at least 6-fold more potent in the antagonist readout were considered active, compounds with an absolute curve rank <1 were considered inactive, and inconclusive otherwise.

### 2.5 Quantitative reverse transcriptase polymerase chain reaction (qRT-PCR)

Fully differentiated NoSpin™ HepaRG™ cells (Lonza, Walkersville, Maryland) were seeded into 12-well collagen-coated plates (Corning) at a density of 8 × 10^5^ cells/well using Williams’ E medium (ThermoFisher) supplemented with HepaRG™ thawing and plating medium supplement (Lonza), 25 U/mL penicillin, and 25 μg/mL streptomycin (Invitrogen). The cells were then placed into a 37°C and 5% CO_2_ incubator for 3 days including a media change before treatment. Cells were then treated with the vehicle control (final concentration of 0.1% DMSO), the positive controls, or test compounds for 24 h before harvesting.

Total RNA was extracted from the cell lysate using an RNeasy Mini kit (Qiagen) according to the manufacturer’s instructions. RNA was reverse transcribed to cDNA using a High-Capacity RNA-to-cDNA kit (Applied Biosystems). CYP3A4 and CYP2B6 mRNA expression were both measured and normalized to the glyceraldehyde-3-phosphate dehydrogenase (GAPDH) housekeeping gene. Real-time PCR was completed using a QuantStudio Real-Time PCR system (Applied Biosystems) with TaqMan probes (CYP3A4 Assay ID: Hs00604506_m1, CYP2B6 Assay ID: Hs04183483_g1, GAPDH Assay ID: Hs99999905_m1, ThermoFisher Scientific, Waltham, MA). Following amplification, induction values were calculated using the equation: Fold = 2^−ΔΔCt^, where ΔCt represents the difference in number of cycles required to reach the Ct threshold between CYP3A4 or CYP2B6 and GAPDH, and ΔΔCt represents the relative change in these differences between the control (DMSO) and treatment groups. Experimental data are expressed as a mean of triplicate values ±SD. Statistical analysis was performed using one-way analysis of variance with *post hoc* Dunnett’s analysis. The statistical significance values were set at *p* < 0.05 (*), *p* < 0.01 (**), and *p* < 0.001 (***).

### 2.6 Molecular docking

Docking of potential antagonists fusidic acid, GSM2, JNJ-31020028, PU-H71, and UVI3003 to the human PXR (hPXR) ligand binding domain (LBD) was performed using AutoDock Vina (Trott and Olson, 2010). The crystal structure of hPXR complexed with SJB7, was retrieved from the protein data bank (PDB ID: 5X0R). The 3D conformers of the ligands were downloaded from PubChem. The protein and ligand pdbqt files needed for docking were created in AutoDock Tools. A grid box that covers the binding pocket site of the protein at the XYZ dimensions was set to 40 Å × 40 Å x 40 Å, with co-ordinates of x: 30.513, y: 15.518, and z: 24.695. Analysis and visualization of the docking results was performed in PyMOL.

## 3 Results

### 3.1 Quantitative high-throughput screen performance

HepG2-*CYP3A4*-hPXR cells were used to perform a primary screen of the NPACT library to identify PXR antagonists. The positive control, SPA70, displayed a consistent IC_50_ throughout the screen, consisting of 35 plates, equaling 0.169 ± 0.029 µM. The screening statistics, showing an acceptable screen performance, are as follows: coefficient of variance (CV): 7.04% ± 1.38; background-to-signal (B/S) ratio: 3.54 ± 0.391; and Z′ factor: 0.572 ± 0.071.

### 3.2 Identification of PXR antagonists

**FIGURE 1:**
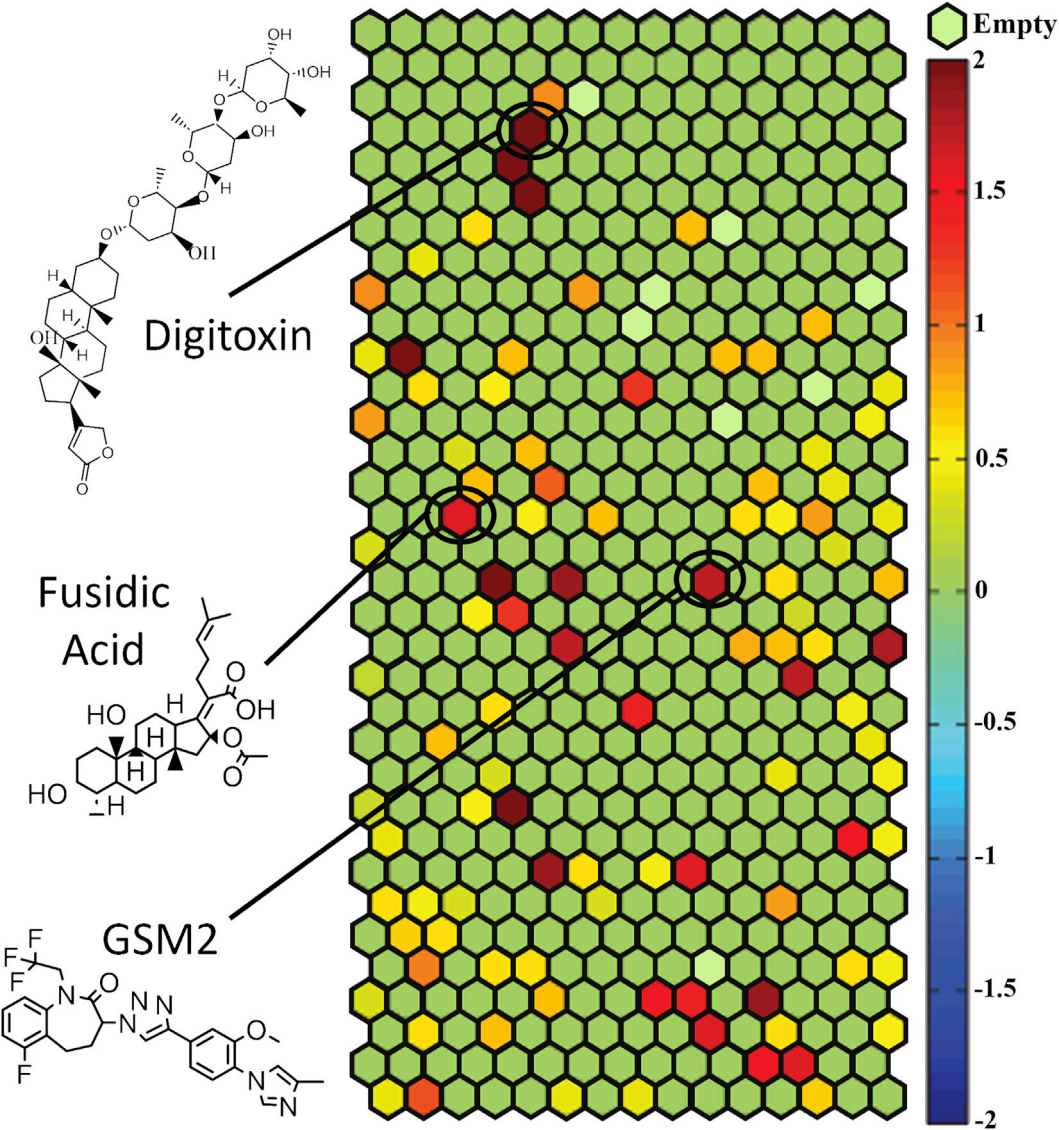
HepG2-*CYP3A4*-hPXR cells were used to screen the NPACT library and compounds were divided into structural clusters. Each hexagon represents a class of structurally similar compounds. The color gradient is indicative of the enrichment of PXR antagonist actives in that specific cluster (negative logarithmic scale of the *p*-value, −log [*p*-value]). Each color represents a group of chemicals with similar scaffolds to antagonize PXR. Clusters with multiple actives in their class are closer to a maroon color, whereas clusters with no activity are a green color. Empty clusters with no available (N/A) compounds in them are light green in color. For important clusters, the structure of an important compound in that cluster is shown.

Once the primary screen of the NPACT library was complete, compounds were clustered based on structural similarity using LeadScope^®^ fingerprints by applying the self-organizing map (SOM) algorithm (Kohonen, 2006). Each cluster was evaluated for the enrichment of active hPXR antagonists by comparing the percentage of active compounds within the cluster with the library average (Figure 1). Statistical significance was determined by the Fisher’s exact test. In this study, we identified 22 structural clusters (colored in maroon in Figure 1) with a statistically significant (*p* < 0.05) enrichment of potential hPXR antagonists. Many of the clusters have been shown to interact with PXR previously, such as the terpene lactones (k11.2), steroid lactones (k6.5), structurally similar heat shock protein 90 inhibitors (k27.6), and benzoic acids (k18.4). One of the clusters including cardiac glycosides (k.4.5), such as digoxin and digitoxin, has been identified as enriched with PXR antagonists from this screening. Digoxin has been well-characterized and implicated in certain drug-drug interactions (DDIs) caused by hPXR modulation in clinical settings (Nicolussi et al., 2020). However, to our knowledge, no such information has been documented about the potential effects of digitoxin modulating hPXR until this study; all the compounds identified within these clusters warrant further studies to rule out toxic side effects, such as DDIs.

**FIGURE 2:**
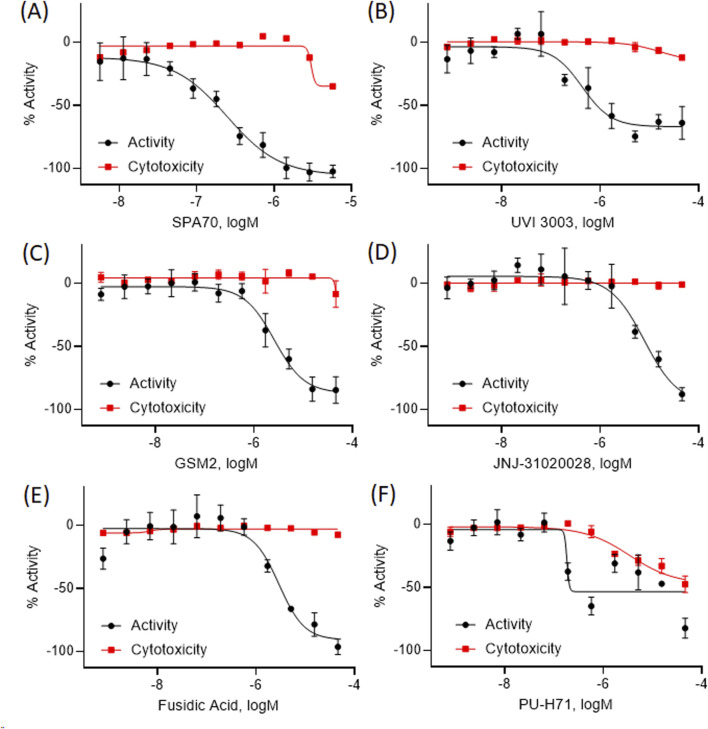
The concentration response curves for five representative compounds with hPXR antagonism potentials are shown here **(B–F)** alongside the positive control **(A)**. The black curve is the hPXR antagonist activity, while the red curve shows the cytotoxicity curves for each compound. All data were compared to the positive control, SPA70 (100% = SPA70 activity) for the activity curves, or tetraoctylammonium bromide (100% = tetraoctylammonium bromide) for the cytotoxicity curves. Data were expressed as mean ± SD from triplicate experiments.

Out of the 5,099 compounds tested from the entire NPACT library, 140 compounds were chosen for a confirmation screen based on efficacy (E < −50%), potency (IC_50_ < 10 µM), a greater than 6-fold difference between activity and viability to suggest no toxicity, or as a negative control. These compounds were rescreened using the same HepG2- *CYP3A4*-hPXR cell line as the primary screen and a 70.2% confirmation rate was found among the active antagonists. Based on efficacy (E < −75%), potency (IC_50_ < 10 µM), and no toxicity, 20 compounds were selected for further in-depth follow-up studies. The curves for the final five compounds are shown in Figure 2, as a representation of this data.

### 3.3 CYP3A4 mRNA inhibition in HepaRG cells

**FIGURE 3:**
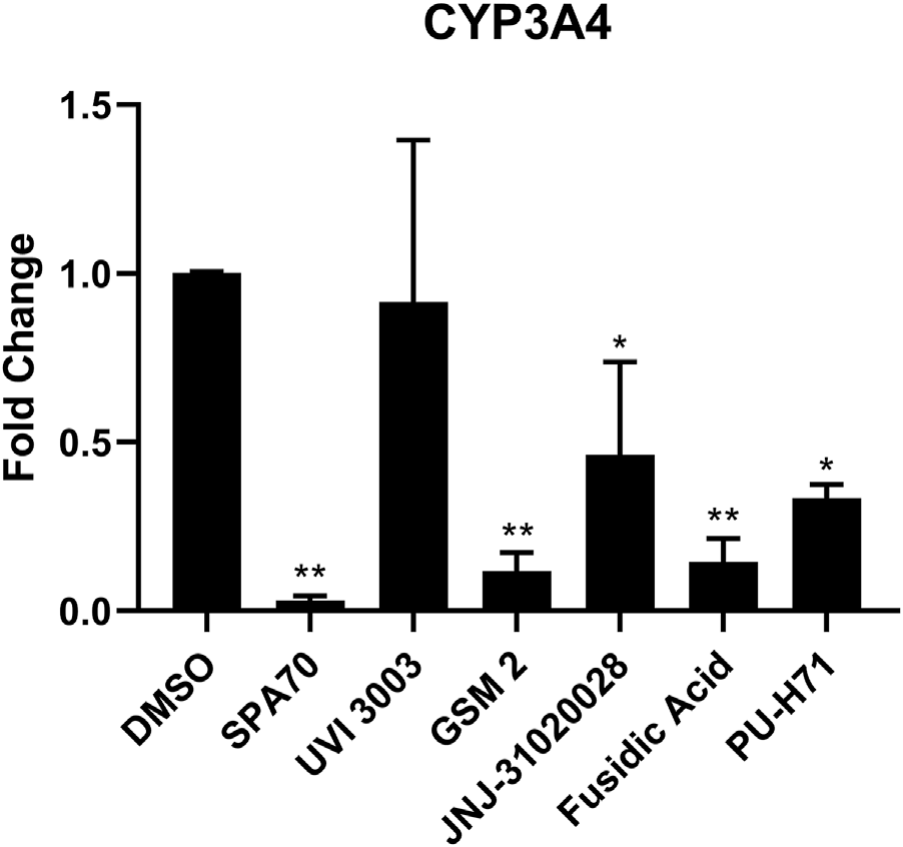
HepaRG cells were treated with the top 20 most promising PXR antagonists identified from the high-throughput screen. The five compounds which showed inhibition of CYP3A4 mRNA levels are shown here (UVI 3003, 0.8 µM; GSM2, 4 μM; JNJ-31020028, 10 μM; fusidic acid, 5 μM; and PU-H71, 0.5 µM), alongside the positive control (SPA70, 2.2 µM) and vehicle control (DMSO, 0.1%). The concentrations for the test compounds were the IC_70_ values from the original NPACT screen. Real-time PCR was used to analyze the mRNA expression of CYP3A4. Each bar represents the mean ± SD in triplicate. *, *p* < 0.05; **, *p* < 0.01.

Although immortalized liver cell lines (i.e., HepG2) are easily adapted into a high-throughput screen (HTS) platform, they are missing many metabolic components, such as nuclear receptors and drug metabolizing enzymes, and therefore do not model a physiologically relevant hepatocyte system (Gerets et al., 2012). Recently, a cryopreserved, predifferentiated form of HepaRG cells were shown to be a promising alternative to human primary hepatocytes by revealing comparable results (Jackson et al., 2016). To overcome the previous screening limitations, we used this more physiologically relevant cell line, HepaRG cells, to run follow-up studies with the PXR antagonists identified from the primary screen which used immortalized cell lines without a metabolic component. Using HepaRG cells, the 20 potential PXR antagonists were treated for 24 h and harvested for mRNA (Supplementary Figure S1). Out of the 20 compounds, 5 compounds were found to inhibit CYP3A4 expression with 4 of them showing significant inhibition (Figure 3).

### 3.4 Pharmacological inhibition curves of PXR in HepG2-*CYP3A4*-hPXR cells

**FIGURE 4:**
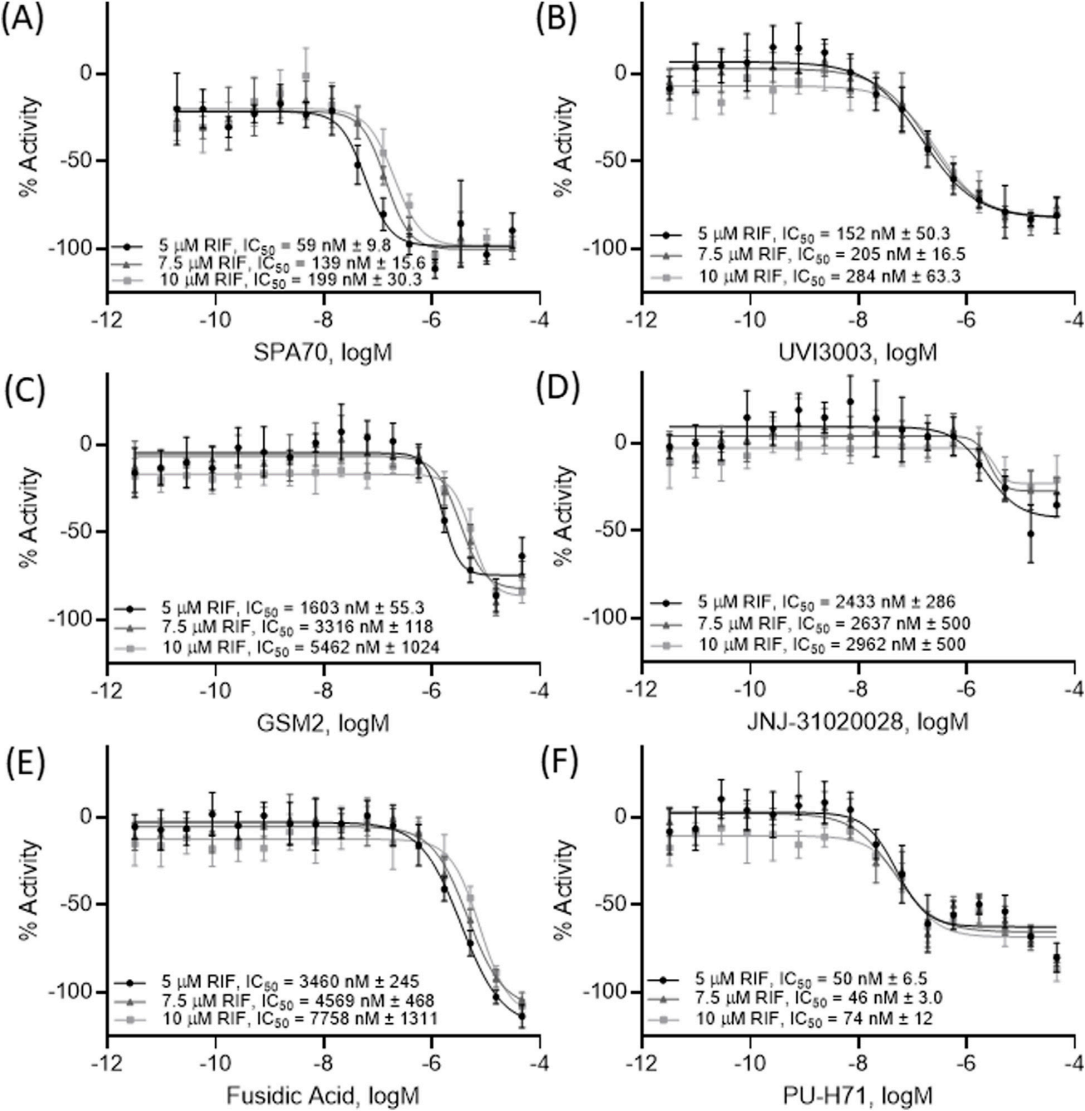
A pharmacological shift assay was performed using HepG2-*CYP3A4*-hPXR cells and concentration response curves were acquired on the 5 compounds **(B–F)** which inhibited CYP3A4 mRNA levels in HepaRG cells as well as the positive control SPA70 **(A)**. Each curve was co-treated with 5 μM RIF, 7.5 μM RIF, or 10 μM RIF (a well-known PXR agonist) to determine pharmacological activity. All data were compared to the positive control, SPA70 (100% = SPA70 activity). The IC_50_, expressed as mean ± SD from triplicate experiments, for each treatment is listed in the figure. Each curve was generated from a combination of all data points together.

To further confirm the top 20 potential PXR antagonists, a pharmacological inhibition assay was performed in the HepG2- *CYP3A4*-hPXR cell line with a co-treatment of varying concentrations of rifampicin, a known PXR activator. If a compound is a true PXR antagonist, the compound’s concentration curve will shift to the right, creating a less potent IC_50_, in the presence of various concentrations of rifampicin. The positive control, SPA70, and 2 of the 4 compounds, GSM2 and fusidic acid, that significantly inhibited CYP3A4 mRNA in HepaRG cells, displayed a more than 2-fold decrease in potency when being co-treated with 2 µM *versus* 10 µM rifampicin (Figure 4).

### 3.5 Inhibitory effects of potential PXR antagonists on CYP3A4 mRNA expression in HepaRG cells

**FIGURE 5:**
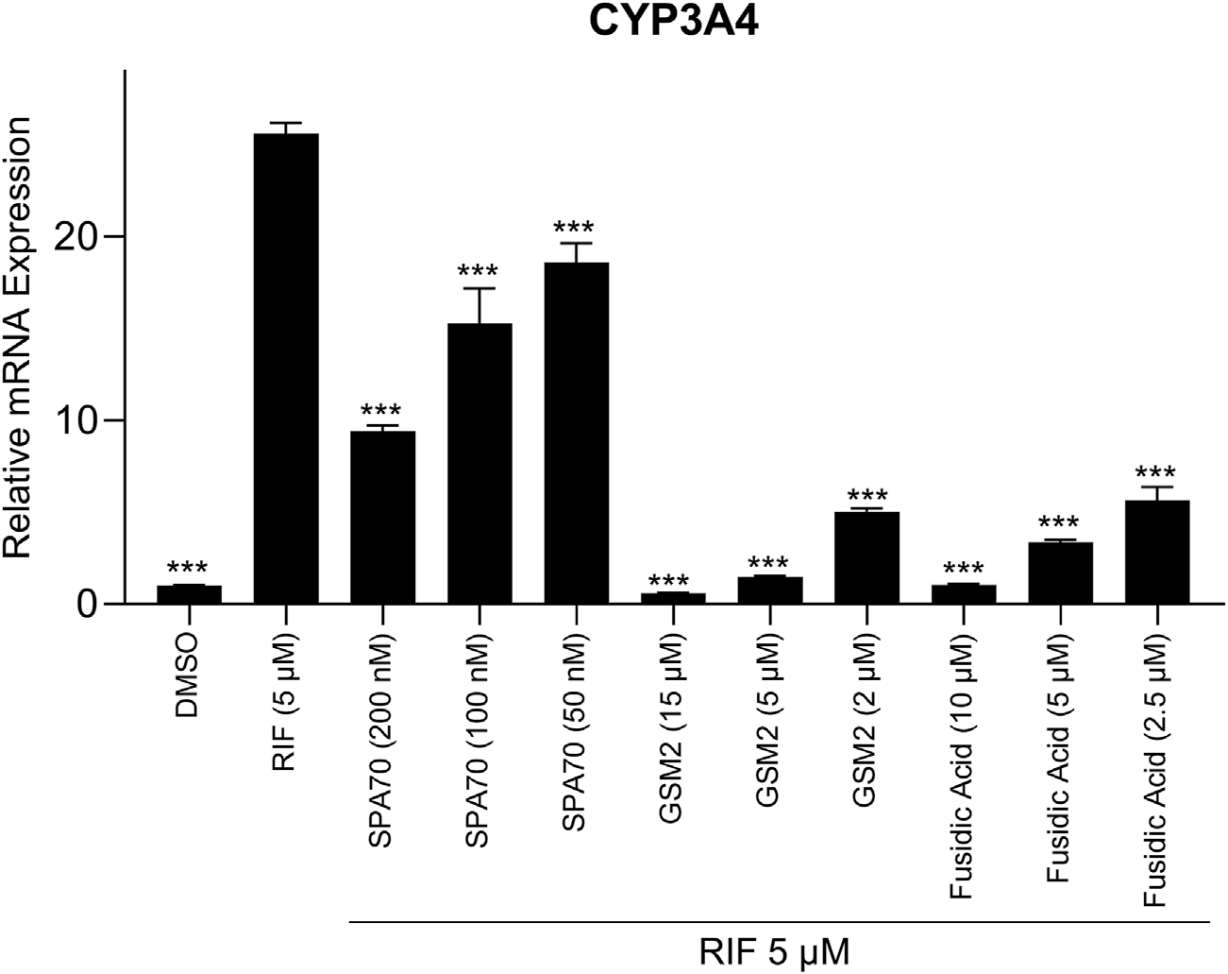
A co-treatment pharmacological assay was performed using HepaRG cells treated with IC_50_, IC_70_, or IC_90_ concentrations of the positive control, SPA70, alongside the two most promising PXR antagonists, GSM2 and fusidic acid. A co-treatment of 5 μM RIF was also in each well to determine if the compound could decrease CYP3A4 mRNA expression once treated with a PXR agonist. DMSO (0.1%) and RIF (5 µM) were also treated in separate wells to determine negative and positive controls, respectively. Real-time PCR was used to analyze the mRNA expression of CYP3A4. Each bar represents the mean ± SD in triplicate. ***, *p* < 0.001.

As previously mentioned, HepG2 cells are not metabolically competent and therefore use of HepaRG cells or human primary hepatocytes is a better choice to confirm PXR antagonists. Therefore, to verify that GSM2 and fusidic acid are indeed PXR antagonists, a smaller scale confirmation study was performed. This study assessed CYP3A4 mRNA expression in metabolically competent HepaRG cells that were treated with both 5 μM rifampicin and each potential antagonist’s IC_50_, IC_70_, or IC_90_ concentration (Figure 5). From the study, GSM2 and fusidic acid both demonstrated a clear concentration dependent inhibition of CYP3A4 mRNA expression. At their highest concentrations, both compounds acquired DSMO levels of CYP3A4 mRNA expression, demonstrating the ability of each compound to completely negate rifampicin’s activation of the PXR pathway. GSM2 was able to inhibit CYP3A4 mRNA expression further than the base level, DMSO’s value of 1, at its highest concentration of 15 μM with an average level of 0.6-fold.

### 3.6 Docking analysis

**FIGURE 6:**
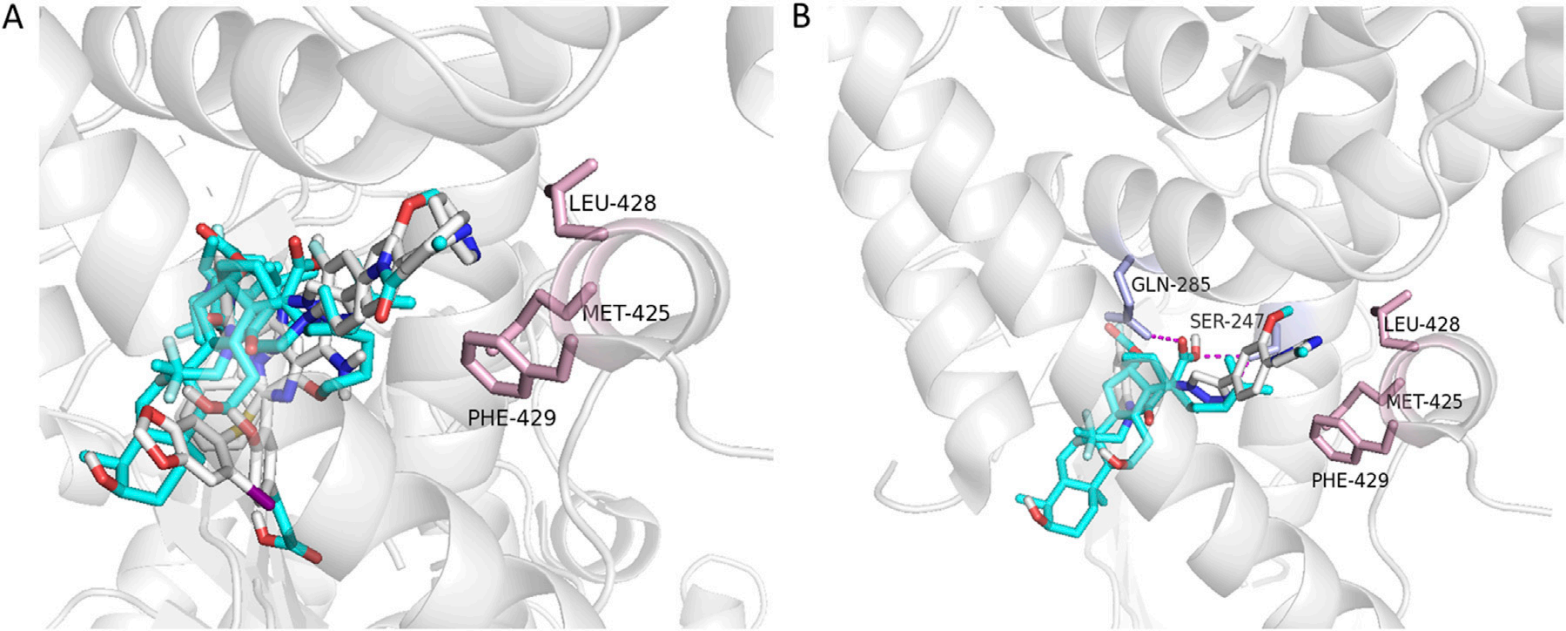
Docking of potential antagonists to the hPXR LBD. The five potential antagonists, fusidic acid, GSM2, JNJ-31020028, PU-H71, and UVI3003 (shown as sticks color coded for different elements of the ligands), were docked in the ligand-binding site **(A)**. Fusidic acid and GSM2 displayed hydrogen-bond interactions (shown as dashed lines in magenta) with SER-247 and GLN-285, and no interactions observed with residues MET-425, LEU-428, and PHE-429 (shown as sticks in light pink from the AF-2 helix of hPXR) **(B)**.

To understand the mode of action of the potential antagonists reported in this study, computational docking was performed. While the co-crystal structure of hPXR with an hPXR antagonist has not yet been reported, a previous study utilized differential hydrogen deuterium exchange mass spectrometry (HDX-MS) to analyze protein-ligand interactions. This study demonstrated that the known PXR antagonist (SPA70) binds directly to the ligand-binding pocket, similar to where the SJB7 (an hPXR agonist) binds (Lin et al., 2017). Additionally, SJB7, an analog of SPA70, was shown to interact with residues such as MET-425, LEU-428, and PHE-429 in the AF-2 helix, and these selective interactions favor agonism (Lin et al., 2017; Li et al., 2021). In the current study, potential antagonists were shown to dock in the ligand binding site (Figure 6A) and interact with key residues, such as SER-247 and GLN-285 (Figure 6B). Additionally, these compounds did not interact with residues in the AF-2 helix, thereby preventing coactivator interaction. Docking analysis has shown that the lack of interactions with residues MET-425, LEU-428, and PHE-429 prevents these compounds from stabilizing the AF-2 helix for co-activator binding, which supports the fact that these compounds exhibit potential antagonistic activity. However, this data does not display direct PXR binding and future studies are needed to determine binding activity.

## 4 Discussion

Identification of PXR antagonists has recently become an important part of compound profiling, due to the varying functions this nuclear receptor exhibits. As previously stated, PXR is a transcription factor which regulates important DMEs, and is also involved in energy homeostasis, cancer, and immune response. The current study identified two pharmaceutical drugs, GSM2 and fusidic acid, as potentially novel PXR antagonists. Due to their significant inhibition of CYP3A4, it is crucial that further studies be completed on any compound that is metabolized by this important DME so that severe side effects do not occur if taken as a co-treatment.

In this study, the NPACT library was screened using a double stable HepG2 cell line including PXR and CYP3A4-luc promoter regions (Lin et al., 2008). Twenty-four structural clusters were identified to be enriched with PXR antagonists (Figure 1). Individual compounds were also chosen alongside representative compounds from these clusters for a follow-up screen of 140 possible actives. Twenty potential PXR antagonists were selected from the primary and follow-up screens for further studies based on potency (IC_50_ < 10 µM), efficacy (E < −50%), cytotoxicity, novelty, and cost to confirm their inhibition of PXR. CYP3A4 mRNA expression was determined using quantitative reverse-transcriptase polymerase chain reaction (qRT-PCR) in HepaRG cells (a physiologically relevant system to human primary hepatocytes (Gerets et al., 2012; Jackson et al., 2016)) for these 20 compounds, and four of them, GSM2, JNJ-31020028, fusidic acid, and PU-H71, significantly inhibited CYP3A4 mRNA expression (Figure 3). A fifth compound, UVI 3003, also generated CYP3A4 inhibition but not to a significant degree. A pharmacological inhibition assay was then performed using the HepG2-*CYP3A4*-PXR cell line, co-treating a concentration response curve of each compound with 5 μM, 7.5 µM, or 10 µM of rifampicin, the well-known PXR agonist. Only two compounds, GSM2 and fusidic acid, generated a shift in IC_50_ values indicating the involvement of PXR in their CYP3A4 inhibition pathway. This finding was also confirmed using HepaRG cells by co-treating increasing concentrations of compound with 5 µM of rifampicin and acquiring a concentration response inhibition of CYP3A4.

The neurodegenerative disorder Alzheimer’s disease is thought to mainly be caused by an abnormal aggregation of amyloid-β (Aβ) plaques in brain cells (Hakem et al., 2024). Modulating the γ-secretase complex has become a recent target in treating this progressive disease. Specifically, Aβ42 has been identified as a neurotoxic species leading to cell death and eventual cognitive impairment in animals (Hung et al., 2008; Moreno et al., 2009; Abramowski et al., 2012). Fischer et al. (2015) identified a novel triazolobenzazepinone, GSM2 (compound 34), as a target to lower cerebral spinal fluid levels of Aβ42 and is potentially going to be considered as a treatment for Alzheimer’s disease (Nordvall et al., 2023). Here, this compound was identified as a PXR antagonist, implying there could be DDIs occurring when co-treating patients with any drug metabolized by CYP3A4. Caution should be used, and more studies need to be completed, to determine the full effects of GSM2 with any other supplement or treatment.

Fusidic acid has been identified as an anti-staphylococcal, antibiotic, and antimicrobial (Wu et al., 2018; Gomaa et al., 2024). Also, there have been substantial studies done to display fusidic acid’s effects as an inhibitor of inflammation induced by bacterial infection (Wu et al., 2018). Therefore, this drug has become an important treatment for bacterial infections and should now be studied completely with respect to its many observed DDIs across different drug classes (Gupta et al., 2016). These include HIV protease inhibitors (like ritonavir and saquinavir), statins (such as atorvastatin and simvastatin), poly ADP ribose polymerase (PARP) inhibitors (like niraparib), and notably, antibiotics such as rifampicin (Khaliq et al., 2000; Herring et al., 2009; Marsot et al., 2017; Damerval et al., 2023). Rifampicin, the previously mentioned and known PXR activator, is occasionally used in combination with oral fusidic acid to treat MRSA-mediated bone and joint infections (Aboltins et al., 2007; Wang et al., 2012). Interestingly, it has been established that this treatment leads to DDI with decreased rifampicin clearance and potentially supratherapeutic levels of rifampicin (Bel, Bourguignon et al., 2017). Given fusidic acid has now been identified, from this study, as a PXR antagonist, it is possible that there is a complex bidirectional modulation of the PXR pathway between fusidic acid and rifampicin that leads to this important DDI and therefore, more work needs to be done to elucidate the mechanism of action occurring. Considering fusidic acid’s clinical history of DDIs and its now confirmed PXR antagonistic properties, there is an implication that any compound metabolized by CYP3A4 and co-treated with fusidic acid needs further study and should be prescribed carefully.

In conclusion, gamma-secretase modulator 2 and fusidic acid were identified as newly characterized PXR antagonists through screening the NPACT library utilizing HepG2-*CYP3A4*-hPXR cells. These cells were also used in a pharmacological shift assay to confirm PXR antagonistic activity. HepaRG cells, a more metabolically competent cell line, were treated with potential compounds and qRT-PCR was performed to confirm inhibition of PXR’s main DME, CYP3A4. Antagonizing PXR could be used therapeutically or have an impact on cell proliferation, metastasis, apoptosis, and energy homeostasis within cancer cells due to its expanding role. DDIs are also a key motive in identifying PXR antagonists, as they will inhibit CYP3A4 activity, decreasing metabolism of any drug connected to this important DME. This initial study requires follow-up steps to be conducted when co-treating compounds with GSM2 and fusidic acid for a more complete model of physiological activity in the human body.

## Data Availability

The original contributions presented in the study are included in the article/Supplementary Material, further inquiries can be directed to the corresponding author.
