# Supplementary material for: Identification of human pregnane X receptor antagonists utilizing a high-throughput screening platform
Source: Front Pharmacol. 2024 Oct 23;15:1448744. doi: 10.3389/fphar.2024.1448744 (PMC11537999; doi:10.3389/fphar.2024.1448744)
Supplement: Supplementary file 1 [file Image1.pdf]

Supplementary Material

1 Supplementary Figures and Tables

1.1 Supplementary Figures

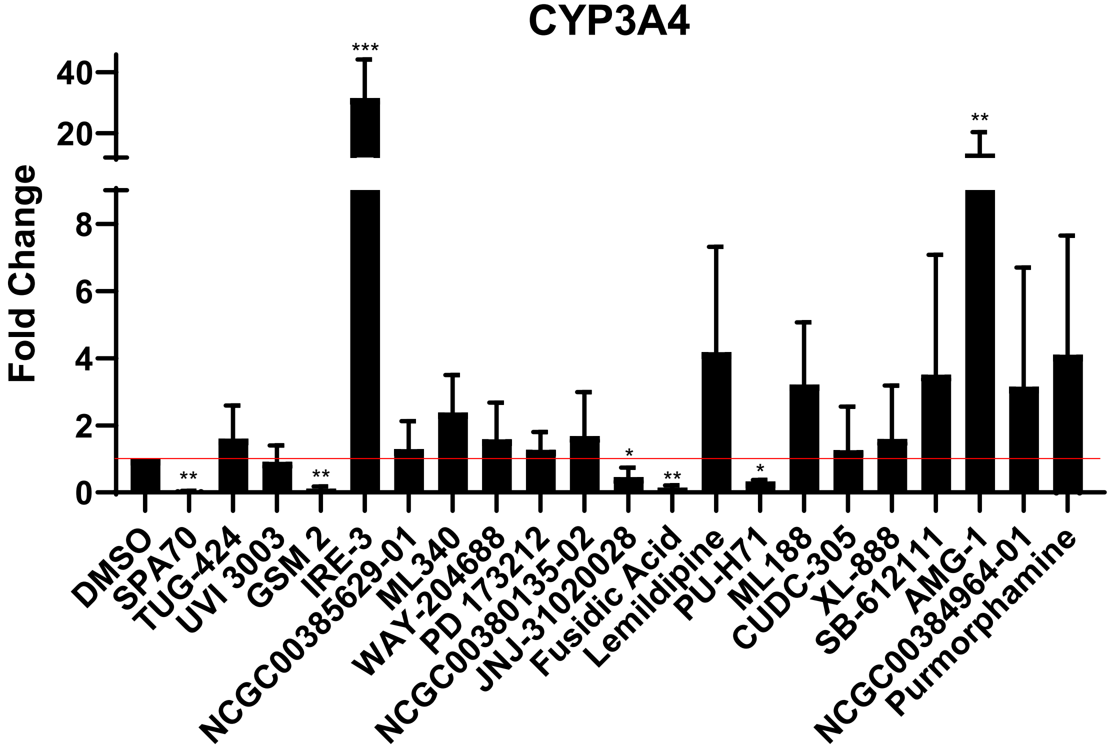

Supplementary Figure 1. HepaRG cells were treated with the top 20 most promising PXR antagonists identified from the high-throughput screen. The concentrations for the test compounds were the IC_{70} values from the original NPACT library screen and are as follows: TUG-424, 3 μM; UVI 3003, 0.8 μM; GSM2, 4 μM; IRE-3, 10 μM; NCGC00385629-01, 0.8 μM; ML 340, 3 μM; WAY-204688, 2 μM; PD 173212, 3 μM; NCGC00380135-02, 7 μM; JNJ-31020028, 10 μM;

fusidic acid, 5 μM; lemildipine, 0.6 μM; PU-H71, 0.5 μM; ML 188, 1 μM; CUDC-305, 0.25 μM; XL-888, 0.5 μM; SB-612111, 8 μM; AMG-1, 20 μM; NCGC00384964-01, 15 μM; and purmorpharmine, 10 μM. The positive control, SPA70, was treated at 2.2 μM and the vehicle control (DMSO) at 0.1%. Real-time PCR was used to analyze the mRNA expression of CYP3A4. Each bar represents the mean ± SD in triplicate. *, $p < 0.05$; **, $p < 0.01$; *** < 0.001.
